# Fatty Acids and Inflammatory Protein Biomarkers From Coronavirus Disease 2019 Patients

**DOI:** 10.1002/iid3.70218

**Published:** 2025-07-08

**Authors:** Niharika Bala, Alaa H. Habib, Marianne Kozuch, Nancy D. Denslow, Neha S. Dhaliwal, Anna H. Owings, Sarah C. Glover, Abdel A. Alli

**Affiliations:** ^1^ Department of Physiology and Aging University of Florida College of Medicine Gainesville Florida USA; ^2^ Department of Physiology, Faculty of Medicine King Abdulaziz University Jeddah 21589 Saudi Arabia; ^3^ Department of Physiological Sciences and Center for Environmental and Human Toxicology University of Florida Gainesville Florida USA; ^4^ Department of Medicine Division of Digestive Diseases, University of Mississippi Medical Center Jackson Mississippi USA; ^5^ Department of Medicine Tulane University School of Medicine New Orleans Louisiana USA; ^6^ Department of Medicine Division of Nephrology, Hypertension, and Renal Transplantation, University of Florida College of Medicine Gainesville Florida USA

**Keywords:** chemokines, COVID‐19, cytokines, fatty acids, inflammation, SARS‐CoV‐2

## Abstract

**Background:**

The coronavirus disease 2019 (COVID‐19) is a viral infection caused by severe acute respiratory syndrome coronavirus 2 (SARS‐CoV‐2) and associated with systemic inflammation. Inflammation is an important process that follows infection and facilitates the body's innate immune response and repair of damaged tissue. Polyunsaturated fatty acids play an important role in the inflammatory process. These lipids can target transcription factors to modulate gene expression and protein function.

**Methods:**

Here, we performed a fatty acid methyl ester (FAME) analysis and immunoassays to evaluate whether differences in basal levels of different types of biomarkers can be detected in freshly frozen plasma samples from patients with and without COVID‐19.

**Results:**

FAME analysis showed a decrease in arachidic acid and myristic acid, but an increase in caprylic acid, palmitic acid, and eicosenoic acid in the plasma of COVID‐19 patients compared to non‐COVID‐19 patients. Multiple chemokines including IP‐10, MCP‐1, and MIP‐1 beta were increased in the COVID‐19 group compared to the non‐COVID‐19 group. Similarly, cytokines including IL‐1 alpha and IL‐8, and cell adhesion and inflammatory response markers including ICAM‐1 and E‐selectin were greater in the plasma of COVID‐19 patients compared to non‐COVID‐19 patients.

**Conclusions:**

A baseline signature of specific polyunsaturated fatty acids, cytokines, and chemokines present in the plasma after COVID‐19 viral infection may serve as biomarkers that can be useful in various applications including determination of severity of infection, indication of disease prognosis, and consideration for therapeutic options.

## Graphical abstract

The coronavirus disease 2019 (COVID‐19) pandemic created the need to identify new biomarkers and drug targets. In this study, we aimed to establish a unique signature profile of specific fatty acids, cytokines, and chemokines present in the plasma after COVID‐19 viral infection to serve as novel biomarkers that can be used to determine the severity of infection, disease prognosis, and consideration for therapeutic options.

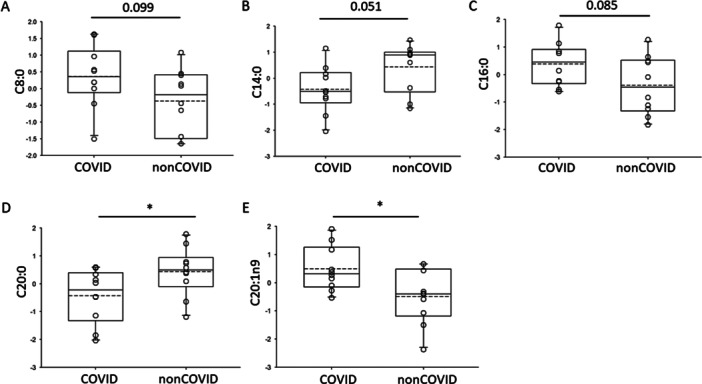

## 1 Introduction

Fatty acids play a key role in signal transduction and in the production of mediators involved in inflammatory pathways associated with viral infection. Polyunsaturated fatty acids are known to regulate lipid raft formation associated with SARS‐CoV‐2 entry mechanisms including transmembrane protease serine‐2 (TMPRSS2) and angiotensin‐converting enzyme‐2 (ACE2) [1]. The overall effect of an inflammatory response is dependent on the interplay between pro‐ and anti‐inflammatory mediators. Pro‐inflammatory cytokines are involved in early responses and amplify inflammatory processes, while anti‐inflammatory cytokines have the opposite effect. Several studies have reported that cytokines are released into the bloodstream after severe COVID‐19 and the resulting cytokine storm can be a consequence of different mechanisms. Hirano and Murakami suggested that one possible mechanism of the cytokine storm in COVID‐19 is mediated by nuclear factor‐κB (NF‐κB) activation, reduction in ACE2 expression, and a subsequent increase in angiotensin 2 [2]. Numerous strategies have been employed to control the cytokine storm and improve the prognosis and survival of the patients. These therapies include cytokine antagonists [3, 4] and immunomodulators [5]. There have been clinical studies performed involving immunomodulatory approaches to combat lung inflammation induced by cytokine storm [6]. Additionally, studies involving the use of small‐molecule‐based medications to enhance regulatory T cell (Tregs) activity to mitigate the cytokine storm due to severe viral infection have been described by Dhawan et al. [7].

Previous studies have shown that oxidative stress induced during COVID‐19 is associated with changes in lipid profiles and systemic inflammation [8]. The inability to resolve inflammatory responses and/or the persistent activation of the inflammasome can trigger a cascade of deleterious outcomes on multiple organ systems. Free fatty acids or nonesterified fatty acids are important in human pathophysiology and have been found to positively or negatively correlate with various diseases including obesity [9], insulin resistance [10], diabetes mellitus [11], metabolic syndrome [12], diabetic nephropathy [13], cardiovascular disease [14], and cerebrovascular accident [15].

The primary goal of this study was to first evaluate whether the levels of different types of circulating biomarkers from COVID‐19 patients compared to non‐COVID‐19 patients can be determined from freshly frozen plasma samples. Secondary outcomes of this study were to examine putative correlations between these circulating fatty acids, cytokines, and chemokines in the context of COVID‐19 pathophysiology.

## 2 Materials and Methods

### 2.1 Inclusion and Exclusion Criteria

The inclusion and exclusion criteria for this study were similar to a previous study by Ziegler et al. [16]. Briefly, participants were enrolled from outpatient clinics, medical‐surgical units, intensive care units (ICU), or endoscopy units at the University of Mississippi Medical Center (UMMC) between April and September 2020. The UMMC Institutional Review Board approved the study under IRB#2020‐0065. To qualify for the COVID‐19 group, individuals had to be at least 18 years old, test positive for SARS‐CoV‐2 through a nasopharyngeal PCR swab, present with COVID‐19 symptoms such as fever, chills, cough, shortness of breath, and sore throat, and have a body weight exceeding 110 pounds. The Control group consisted of individuals who were at least 18 years old, had a recent negative SARS‐CoV‐2 test (PCR or rapid antigen), and weighed more than 110 pounds. Table S1 gives the COVID‐19 variant, age, and race of each participant. Informed consent was provided by each participant or their legally authorized representative in both groups. Blood samples were collected by trained healthcare personnel using sodium heparin‐containing tubes. Plasma was obtained by centrifuging the tubes at 1200*g* for 10 min and transferring the supernatant to clean tubes for storage in a −20°C freezer.

### 2.2 Blood Plasma FAME Derivatization

Blood plasma samples were prepared for analysis with a direct derivatization technique as previously described [17]. Briefly, the samples were weighed in round bottom glass tubes, and 14% boron trifluoride in methanol was added (Millipore Sigma, Burlington, MA), before being incubated in a 100°C sand bath for 1 h. After cooling, Optima grade hexanes (Fisher Scientific, Hampton, NH) and ultrapure water (MilliQ, Millipore Sigma) were added to the samples, and the samples were shaken to allow the transfer of FAME into the hexane. Hexane extraction was repeated once, and Na_2_SO_4_ (Fisher Scientific) scavenged any remaining water from the hexane extracts. Sample extracts were reduced to 0.3 mL under a gentle stream of Nitrogen (N_2_) at 35°C and spiked with US‐108N mixed deuterated Polycyclic Aromatic Hydrocarbon (PAH) for use as an internal standard. Fatty acid methyl ester (FAME) analysis was carried out on an Agilent 7890B gas chromatograph (GC) coupled to an Agilent 7000 C triple quad mass spectrometer (MS). Running conditions for the GC were as follows: 1 µL of sample was injected into the GC through a 280°C inlet in splitless mode, and helium carrier gas was maintained at 1.2 mL/min at constant flow. Samples passed through a DB‐FATWAX capillary column (30 m length, 250 µm outer diameter, 0.25 µm film thickness, Agilent). The initial oven temperature was 60°C, which was held for 2 min, after which the temperature was ramped at a rate of 10°C/min to 180°C. After a 5‐min hold time, the temperature was ramped to 250°C. This final temperature was held for 3 min. Chromatographically separated sample components passed into the MS through a 280°C transfer line, and were ionized in EI mode. The MS source temperature was 280°C; Q1 quadrupole temperature was held at 150°C. Sample FAME was detected in full scan mode, and quantitated by comparison to pure FAME calibration dilutions 0.01–5 µg/mL (NuChek Prep, Elysian, MN).

**Table:** 

	C14:0	C16:0	C16:1n9	C18:0	C18:1n9
Sample LOD	0.065	0.065	0.065	0.065	0.065
Sample LOQ	0.509	0.254	0.509	0.509	0.509

### 2.3 Human Chemokine Panel 9 Plex

Human COVID‐19 and non‐COVID‐19 plasma samples were centrifuged at 1000 *g* for 10 min at room temperature. Standards and the reagents were freshly prepared before the experiment. Magplex capture beads were added into each well followed by standards and samples. The assay was performed following the manufacturer's manual (Cat No. EPX090‐15840‐90, ThermoFisher Scientific, USA). A Luminex 200 instrument was used to read the plate followed by data processing in ThermoFisher cloud Procartaplex analysis app. The analytes detected included 6Ckine, GCP‐2, I‐309, MCP‐4, MPIF, MIP‐2 alpha, MIP‐3 beta, TARC, TECK as indicated in Table 1. Sigmaplot 15 software was used to perform a Student's *t*‐test and to plot the data (Table 1). The correlation of these inflammatory protein biomarkers with COVID‐19 and associated comorbidities is given in Table 2.

**Table 1:** List of analytes and respective bead numbers.

Analyte	Bead number
6Ckine	46
GCP‐2	64
I‐309	75
MCP‐4	73
MIP‐2 alpha	56
MIP‐3 beta	74
MPIF	53
TARC	30
TECK	67

**Table 2:** Correlation of inflammatory protein biomarkers with COVID‐19 severity and comorbidities.

Marker	Biomarker for	
CD62E/E‐selectin	Critical disease and increased risk of ICU admission	Mezine et al. [18], Oliva et al. [19]
ICAM‐1	Elevated in patients with mild and severe cases	Tong et al. [20], Syed et al. [21], Bédard‐Matteau et al. [22]
IL‐1	Heightened inflammatory response hallmark of severe COVID‐19	Chang et al. [23], Hawerkamp et al. [24], McElvaney et al. [25]
IL‐8	Found elevated in ICU patients	Chang et al. [23], Masso‐Silva et al. [26], Mazaheri et al. [27], McElvaney et al. [25]
MCP‐1	Thrombosis‐related indicator	Chen et al. [28], Jøntvedt Jørgensen et al. [29], Eichhorn et al. [30]
IP‐10	Thrombosis‐related indicator	Chen et al. [28], Yang et al. [31], Haroun et al. [32], Lev et al. [33]
MIP‐1 beta	Mild to moderate COVID severity marker	Codina et al. [34]
CXCL‐2	Chronic inflammation and fibrosis	Scott et al. [35], Strieter et al. [36]

### 2.4 Human Inflammation Panel 20 Plex

Human COVID‐19 and non‐COVID‐19 plasma samples were centrifuged at 1000 *g* for 10 min at room temperature. Standards and reagents were freshly prepared before the experiment. Magplex capture beads were added into each well followed by standards and samples. The assay was performed following the manufacturer's manual (Cat No. EPX200‐12185‐901, ThermoFisher Scientific, USA). A Luminex 200 instrument was used to read the plate followed by data processing in ThermoFisher cloud Procartaplex analysis app. The cytokines detected included GM‐CSF, IFN alpha, IFN gamma, IL‐1 alpha, IL‐1 beta, IL‐4, IL‐6, IL‐8, IL‐10, IL‐12p70, IL‐13, IL‐17A, TNF alpha. The chemokines detected included IP‐10, MCP‐1, MIP‐1 alpha, MIP‐1 beta, and cell adhesion and inflammatory response markers ICAM‐1, CD62E (E‐selectin), CD62P (P‐Selectin) as noted in Table 3. Sigmaplot 15 software was used to perform a Student's *t*‐test and plot the data.

**Table 3:** List of analytes and respective bead numbers.

Analyte	Bead number
GM‐CSF	44
IFN alpha	48
IFN gamma	43
IL‐1 alpha	62
IL‐1 beta	18
IL‐4	20
IL‐6	25
IL‐8	27
IL‐10	28
IL‐12p70	34
IL‐13	35
IL‐17	36
TNF alpha	45
IP‐10	22
MCP1	51
MIP‐1 alpha	12
MIP‐1 beta	47
ICAM‐1	73
CD62E	77
CD62P	55

## 3 Results

### 3.1 Circulating Levels of Fatty Acids in Patients With or Without COVID‐19

**Figure 1:**
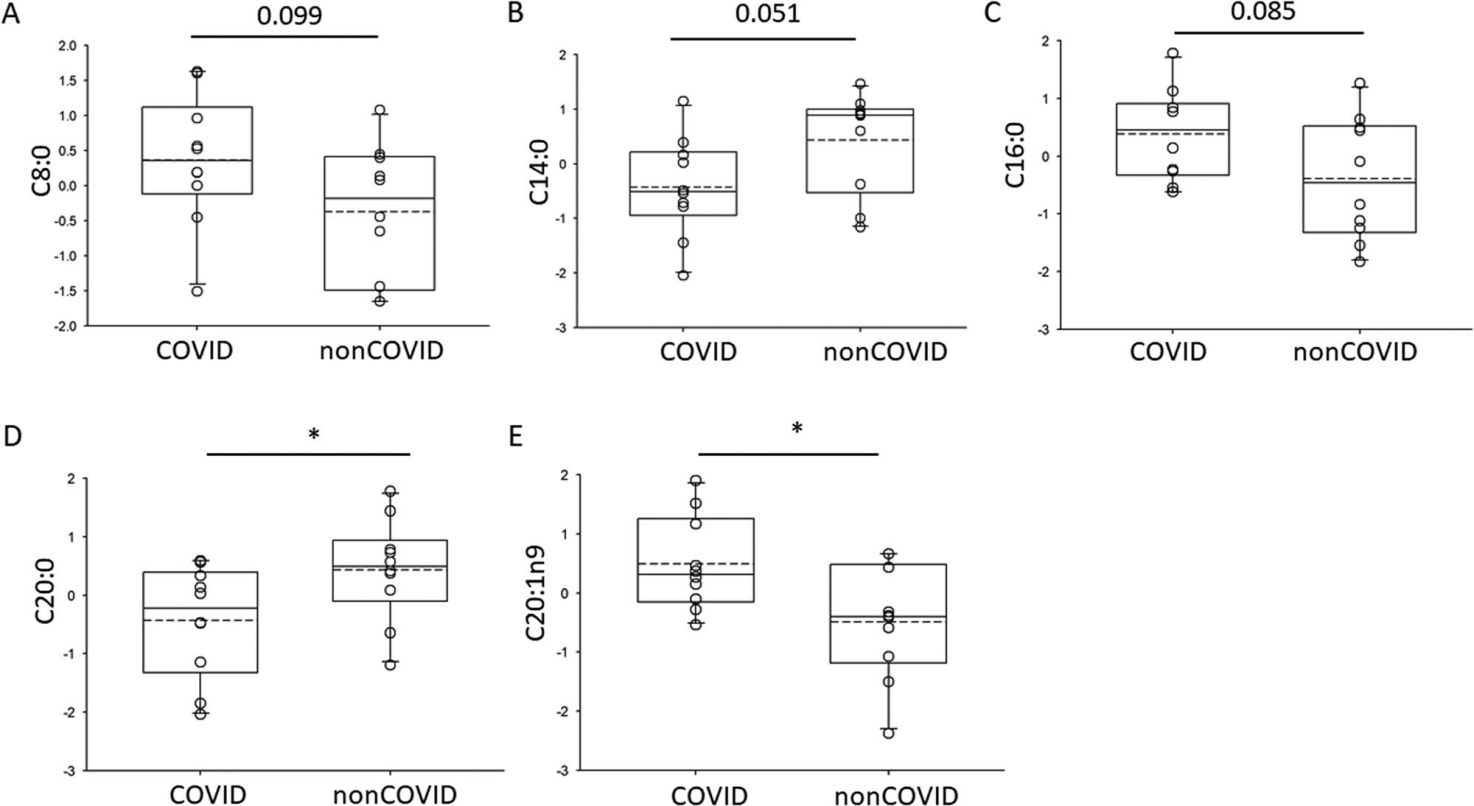
Analysis of free fatty acids in plasma of patients with or without COVID‐19. (A) caprylic acid (C8:0), (B) myristic acid (C14:0), (C) palmitic acid (C16:0), (D) arachidic acid (C20:0), (E) eicosenoic acid (C20:1n9). * represents a *p* < 0.05.

Polyunsaturated fatty acids have been shown to play a role in SARS‐CoV‐2 entry and infection. Therefore, we investigated basal levels of prominent fatty acids in the systemic circulation of COVID‐19 patients compared to non‐COVID‐19 patients. Here, FAME analysis of freshly frozen plasma samples showed higher basal levels of caprylic acid (C8:0), palmitic acid (C16:0), and eicosenoic acid (C20:1n9) in COVID‐19 patients compared to non‐COVID‐19 patients (Figure 1). Conversely, basal levels of myristic acid (C:14:0) and arachidic acid (C20:0) were lower in the COVID‐19 group compared to the non‐COVID‐19 group (Figure 1).

### 3.2 Circulating Levels of Pro‐Inflammatory and Anti‐Inflammatory Cytokines in COVID‐19 and Non‐COVID‐19 Patients

**Figure 2:**
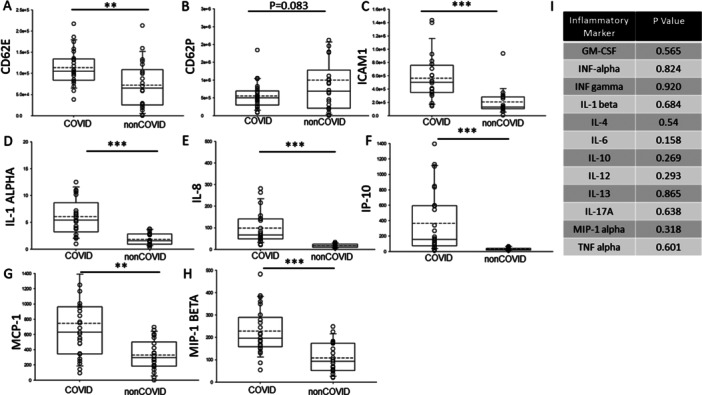
Pro‐ and anti‐inflammatory cytokines detected in the plasma of COVID‐19 and non‐COVID‐19 patients. (A) CD62E, (B) CD62P, (C) ICAM1, (D) IL‐1 alpha, (E) IL‐8, (F) IP‐10, (G) MCP‐1, (H) MIP‐1 beta, (I) table showing inflammatory markers that were comparable between the two groups. ** represents a *p* < 0.01. *** represents a *p* < 0.001.

Pro‐inflammatory cytokines play an important role in early responses and amplify inflammatory reactions while anti‐inflammatory cytokines limit the inflammatory reactions. Therefore, we measured both pro‐inflammatory and anti‐inflammatory cytokines in the plasma of COVID‐19 and non‐COVID‐19 patients. As shown in Figure 2, the levels of CD62E, ICAM1, IL‐1 alpha, IL‐8, IP‐10, MCP‐1, and MIP‐1 beta were augmented in the COVID‐19 group while CD62P was greater in the non‐COVID‐19 group.

### 3.3 Circulating Levels of Chemokines in COVID‐19 and Non‐COVID‐19 Patients

**Figure 3:**
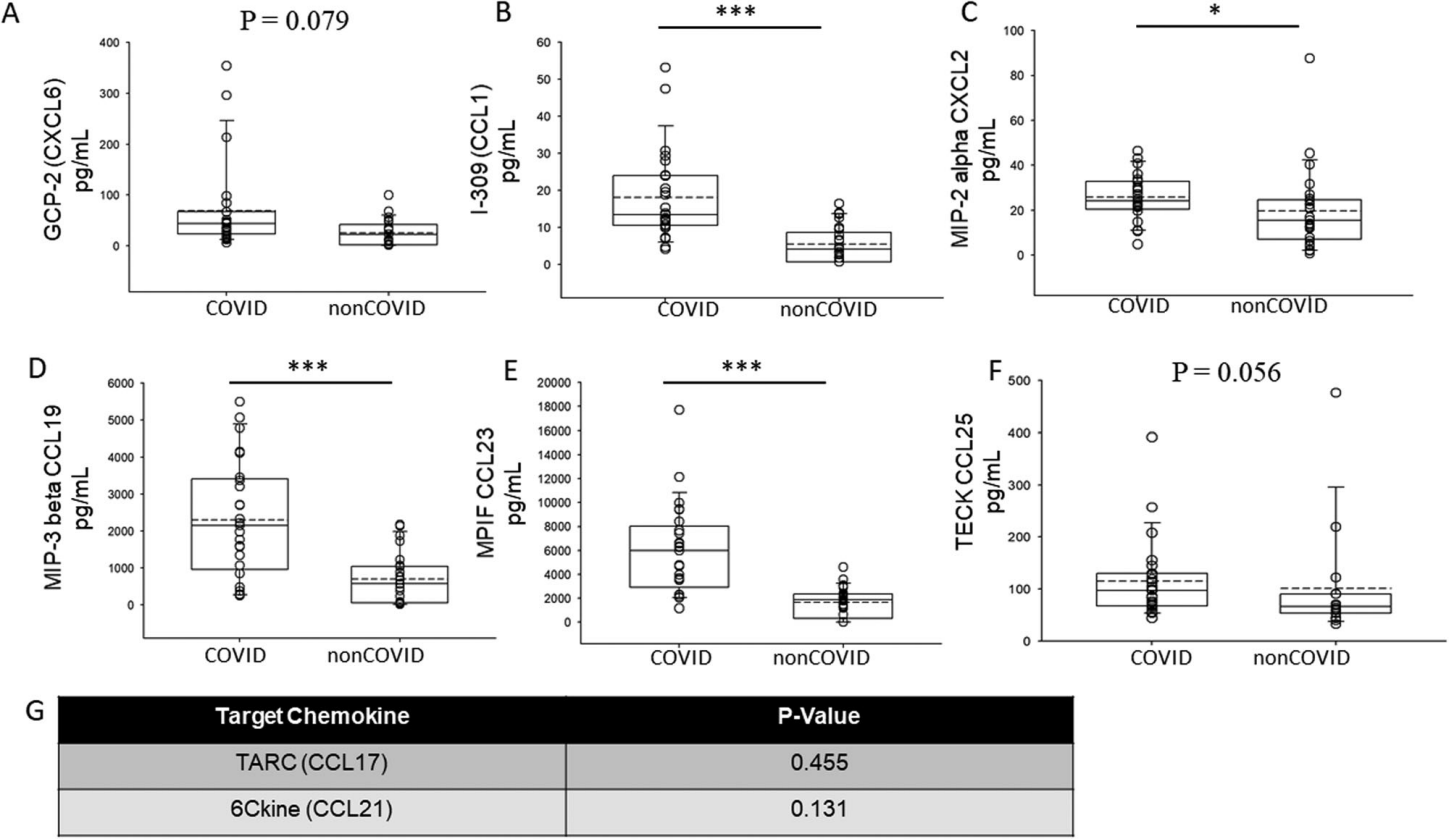
Expression of chemokines in the plasma of COVID‐19 and non‐COVID‐19 patients. (A) GCP‐2, (B) CCL1, (C) MIP‐2 alpha CXCL2, (D) MIP‐3 beta CCL19, (E) MPIF CCL23, (F) TECK CCL25, (G) table showing chemokines that were comparable between the two groups. * represents a *p* < 0.05, ** represents a *p* < 0.01. *** represents a *p* < 0.001.

Chemokines are a class of cytokines that are known to be expressed locally at sites of virally infected tissues [37]. Therefore, we measured the expression of various chemokines in the plasma of COVID‐19 and non‐COVID‐19 patients. As shown in Figure 3, several chemokines including GCP‐2, CCL1, MIP‐2 alpha CXCL2, MIP‐3 beta CCL19, MPIF CCL23, and TECK CCL25 were found to be significantly elevated in the COVID‐19 group compared to the non‐COVID‐19 group.

## 4 Discussion

COVID‐19 remains a public health problem and the long‐term effects are still not known. In addition, there is a need to identify novel biomarkers from the systemic circulation that can be used to predict the severity of infection. Published studies suggest the use of never‐frozen plasma samples offers superior results when compared to the use of freshly frozen plasma samples [38]. In many cases, the recruitment of patients and collection of samples occurs over a period of months and the samples are frozen to be run at a later time. Running samples at the same time offers important advantages that includes achieving a desired sample size per study group, allowing for economic feasibility, and minimizing technical variation. Here, we determined whether statistically significant basal levels of multiple fatty acids, cytokines, and chemokines could be detected in freshly frozen plasma samples from patients with and without COVID‐19.

Fatty acids are important for the construction of complex lipids. These lipids can be synthesized by cells or they can be incorporated from extracellular sources. The quantification of long‐chain fatty acids in samples such as plasma represents a specific class of biomarkers present in the systemic circulation that can provide useful information including elucidating lipid pathways involved in pathophysiology. A study by Spiller and colleagues showed free fatty acids levels are elevated in newly diagnosed and long‐term controlled T2DM patients [11]. Liu and colleagues showed that docosahexaenoic acid negatively correlates with microalbuminuria in diabetic nephropathy [13]. Among the fatty acids that our FAME analysis showed decreased basal levels within the plasma of patients with COVID‐19 compared to patients without COVID‐19 was the 14‐carbon saturated fatty acid, myristic acid. The addition of myristic acid to the amino‐terminal glycine residue of proteins is known to stabilize proteins at the membrane and effect their cellular function. A study by Du and colleagues reported a reduction in myristic acid‐containing dimyristoyl‐PC and palmitoylmyristoyl‐PC in lung surfactant may promote SARS‐CoV‐2 infection and suggested increasing these lipids may be beneficial in treating severe COVID‐19 disease [39]. Importantly, a study by Rampoldi and colleagues showed amino‐terminal myristoylation is crucial for T cell receptor (TCR) signaling as well as the release of cytokines [40]. Another fatty acid that we found to be downregulated in the plasma of COVID‐19 patients compared to non‐COVID‐19 patients was arachidic acid. Fretts and colleagues reported higher levels of circulating arachidic acid are associated with a reduced risk of atrial fibrillation [41]. Circulating arachidic acid has been shown to have beneficial cardiovascular effects [42]. The results from our study suggest that the lower circulating levels of arachidic acid correlate with poor cardiovascular outcomes, which is observed in a large number of COVID‐19 patients. Caprylic acid, palmitic acid, and eicosenoic acid were among the three fatty acids that were found by our FAME analysis to be greater in the plasma of the COVID‐19 group compared to the non‐COVID‐19 group. The increased levels of palmitic acid in the plasma of COVID‐19 patients are consistent with data from published studies. Joshi and colleagues showed palmitic acid contributes to COVID‐19 pathogenesis by modulating molecules involved in the regulation of the immune system and apoptosis [43]. A nutrigenomic analysis study by Barh and colleagues suggested increased severity and higher death rate from COVID‐19 in western countries compared to that in India may be attributed to increased activation of cytokine storm related pathways, as a result of increased consumption of red meat and processed foods products rich in palmitic acid in the former [44]. Presumably, the ability of palmitic acid to induce endoplasmic reticulum stress, as shown by Ma et al. [45], and its ability to induce ACE2 expression in cultured hepatocytes, as shown by Cao et al. [46], may also contribute to increased morbidity and mortality associated with COVID‐19.

Prior studies have investigated the role of cholesterol‐enriched lipid rafts as signaling platforms for the entry of SARS‐CoV‐2 [47]. Lipid rafts do not only serve as binding sites for SARS‐CoV‐2, but they are also involved in the activation and internalization of the virus important for transmission from one cell to another [48]. Our group previously showed that extracellular vesicles isolated from the plasma of COVID‐19 patients increase the density of lipid rafts in human small airway epithelial cells [49]. A study by Teixeira and colleagues showed that the HMG CoA reductase inhibitor, simvastatin, disrupts lipid rafts and inhibits SARS‐CoV‐2 entry into cells and the production of inflammatory cytokines [50]. A study by El Khoury and Naim demonstrated that simvastatin and fluvastatin disrupt the interaction of ACE2 with the lipid rafts and its sorting at the brush border membrane of intestinal epithelial cells [51]. Bailly and Vergoten discussed the role of glycyrrhizic acid in the disorganization of the lipid rafts to inhibit coronavirus entry into cells [52]. A study by Alboni and colleagues showed that depletion of membrane cholesterol from hydroxypropyl‐beta‐cyclodextrin inhibits the entry of SARS‐CoV‐2 into human ACE2 overexpressing cells [53]. Multiple groups have suggested targeting lipid rafts may be effective in the treatment of COVID‐19 as it has been previously used to successfully treat other viral infections [48, 54, 55].

Chemokines play an important role in the recruitment of lymphocytes and leukocytes to injured tissue. They trigger biological activities through the activation of G protein‐coupled chemokine receptors. Some of these receptors recognize multiple chemokines and various chemokines bind to multiple receptors [56]. Liu and colleagues showed CCL1 promotes pulmonary fibrosis development through the activation of the AMFR–Ras–ERK–p70S6K signaling pathway [57]. Our study showed basal levels of multiple chemokines including CCL1 were significantly higher in the plasma of COVID‐19 patients when compared to levels in the plasma of non‐COVID‐19 patients.

Some studies suggest that using freshly obtained and never‐frozen plasma samples are necessary to obtain statistically significant results for the identification of biomarkers. Our study demonstrates that the use of stored plasma samples from patients with or without COVID‐19 can be used to successfully assay different types of biomarkers including unsaturated fatty acids, cytokines, and chemokines. We detected five or more biomarkers from each biomarker type and this data were consistent with expected results while considering similar data from other studies. Limitations of our study include not comparing fresh samples to freshly frozen samples, lack of inclusion of a healthy control group, and not comparing biomarker levels in the plasma from COVID‐19 and non‐COVID‐19 patients at different time points after viral infection. Nevertheless, the data presented here suggest that freshly frozen plasma samples are a rich source of circulating biomarkers that can differentiate between active COVID‐19 viral infection and non‐COVID‐19 disease. Finally, another limitation of our study is that we were unable to group patients based on their comorbidities. It is possible that some patients may have had other diseases that could contribute to the cytokine storm and changes in the different markers we investigated in this study.

Future studies may be performed to determine whether basal levels of these fatty acids, cytokines, and chemokines change in response to vaccination or recovery of COVID‐19. Additionally, it would be interesting to evaluate the levels of these systemic biomarkers in a prospective study aimed at studying the impact of long COVID‐19 associated with a decline in function of a specific organ system.

## Author Contributions

**Niharika Bala:** data curation, formal analysis, investigation, writing – original draft, writing – review and editing. **Alaa H. Habib:** data curation, formal analysis, investigation, resources, writing – original draft, writing – review and editing. **Marianne Kozuch:** formal analysis, investigation, writing – original draft, writing – review and editing. **Nancy D. Denslow:** formal analysis, funding acquisition, supervision, writing – original draft, writing – review and editing. **Neha S. Dhaliwal:** investigation, writing – original draft, writing – review and editing. **Anna H. Owings:** investigation, writing – original draft, writing – review and editing. **Sarah C. Glover:** funding acquisition, resources, supervision, writing – original draft, writing – review and editing. **Abdel A. Alli:** conceptualization, formal analysis, investigation, project administration, resources, supervision, writing – original draft, writing – review and editing.

## Ethics Statement

The UMMC Institutional Review Board approved the study under IRB#2020‐0065.

## Consent

Written informed consent was obtained from all participants or their legally authorized representatives.

## Supporting information

supmat.

## Data Availability

Data sets will be provided upon reasonable request after contacting the corresponding author.

## 1

**Appendix of analytes:**

Arachidic acid (C20:0)

Behenic acid (C22:0)

Capric acid (C10:0)

Caprylic acid (C8:0)

*cis*‐11‐Eicosenoate (C20:1)

Elaidic acid (C18:1n9t)

Erucic (C22:1n9)

Heptadecanoic acid (C17:0)

Lauric acid (C12:0)

Linoleic acid (C18:2n6c)

Linolenic acid (C18:3n3)

Myristic acid (C14:0)

Myristoleic acid (C14:1n9c)

Oleic acid (C18:1n9c)

Palmitic acid (C16:0)

Palmitoleic acid (C16:1n9c)

Pentadecanoic acid (C15:0)

Stearic acid (C18:0)

Tridecanoic acid (C13:0)

C20:1n9 (eicosenoic acid)
