# Supplementary material for: Fatty Acids and Inflammatory Protein Biomarkers From Coronavirus Disease 2019 Patients
Source: Immun Inflamm Dis. 2025 Jul 8;13(7):e70218. doi: 10.1002/iid3.70218 (PMC12235974; doi:10.1002/iid3.70218)
Supplement: Supplementary file 1 — supmat. [file IID3-13-e70218-s001.docx]

**Supplementary Tables.**

| Sample | Gender | Race | Variant |
| --- | --- | --- | --- |
| *COVID-19* |  |  |  |
| C19 1 | Female | African American | Ancestral |
| C19 2 | Female | African American | Ancestral |
| C19 3 | Female | African American | Ancestral |
| C19 4 | Male | Caucasian | Ancestral |
| C19 5 | Male | Caucasian | Ancestral |
| C19 6 | Female | African American | Ancestral |
| C19 7 | Female | Caucasian | Ancestral |
| C19 8 | Male | African American | Ancestral |
| C19 9 | Female | African American | Ancestral |
| C19 10 | Male | Hispanic | Ancestral |
| C19 11 | Male | Caucasian | Ancestral |
| C19 12 | Male | Caucasian | Ancestral |
| C19 13 | Male | Caucasian | Ancestral |
| C19 14 | Female | African American | Ancestral |
| C19 15 | Male | Caucasian | Ancestral |
| C19 16 | Male | African American | Ancestral |
| C19 17 | Male | Caucasian | Ancestral |
| C19 18 | Female | Caucasian | Ancestral |
| C19 19 | Female | Caucasian | Ancestral |
| C19 20 | Female | African American | Ancestral |
| C19 21 | Male | Caucasian | Ancestral |
| C19 22 | Male | Caucasian | Ancestral |
| C19 23 | Male | Caucasian | Ancestral |
| C19 24 | Female | African American | Ancestral |
| C19 25 | Female | African American | Ancestral |
| *nonCOVID-19* |  |  |  |
| NC19 1 | Male | Caucasian | Non COVID-19 |
| NC19 2 | Male | Caucasian | Non COVID-19 |
| NC19 3 | Female | Caucasian | Non COVID-19 |
| NC19 4 | NA | NA | Non COVID-19 |
| NC19 5 | Female | Asian | Non COVID-19 |
| NC19 6 | Female | Caucasian | Non COVID-19 |
| NC19 7 | Female | African American | Non COVID-19 |
| NC19 8 | Female | African American | Non COVID-19 |
| NC19 9 | Male | Caucasian | Non COVID-19 |
| NC19 10 | Male | Caucasian | Non COVID-19 |
| NC19 11 | Female | Caucasian | Non COVID-19 |
| NC19 12 | Female | African American | Non COVID-19 |
| NC19 13 | Male | African American | Non COVID-19 |
| NC19 14 | Female | African American | Non COVID-19 |
| NC19 15 | Female | African American | Non COVID-19 |
| NC19 16 | Male | Caucasian | Non COVID-19 |
| NC19 17 | Male | Caucasian | Non COVID-19 |
| NC19 18 | Female | Caucasian | Non COVID-19 |
| NC19 19 | Female | Caucasian | Non COVID-19 |
| NC19 20 | Female | Caucasian | Non COVID-19 |
| NC19 21 | Male | Caucasian | Non COVID-19 |
| NC19 22 | Female | Caucasian | Non COVID-19 |
| NC19 23 | Female | Caucasian | Non COVID-19 |
| NC19 24 | Female | Caucasian | Non COVID-19 |
| NC19 25 | Female | African American | Non COVID-19 |

Supplementary Table 1. COVID-19 and non-COVID-19 patient information. Reference of gender, ethnicity, and COVID-19 variant. NA refers to not available.

**Appendix of analytes:**

Arachidic acid (C20:0)

Behenic acid (C22:0)

Capric acid (C10:0)

Caprylic acid (C8:0)

cis-11-Eicosenoate (C20:1)

Elaidic acid (C18:1n9t)

Erucic (C22:1n9)

Heptadecanoic acid (C17:0)

Lauric acid (C12:0)

Linoleic acid (C18:2n6c)

Linolenic acid (C18:3n3)

Myristic acid (C14:0)

Myristoleic acid (C14:1n9c)

Oleic acid (C18:1n9c)

Palmitic acid (C16:0)

Palmitoleic acid (C16:1n9c)

Pentadecanoic acid (C15:0)

Stearic acid (C18:0)

Tridecanoic acid (C13:0)

C20:1n9 (eicosenoic acid)
